# Commuting, Life-Satisfaction and Internet Addiction

**DOI:** 10.3390/ijerph14101176

**Published:** 2017-10-05

**Authors:** Bernd Lachmann, Rayna Sariyska, Christopher Kannen, Maria Stavrou, Christian Montag

**Affiliations:** 1 Molecular Psychology, Institute of Psychology and Education, Ulm University, Helmholtzstrasse 8/1, 89081 Ulm, Germany; rayna.sariyska@uni-ulm.de (R.S.); christian.montag@uni-ulm.de (C.M.); 2 Department of Informatics, University of Bonn, 53012 Bonn, Germany; info@ckannen.com; 3 Department of Psychology, Goldsmiths, University of London, London SE14 6NW, UK; mstav010@gold.ac.uk; 4 Key Laboratory for NeuroInformation/Center for Information in Medicine, School of Life Science and Technology, University of Electronic Science and Technology of China, Chengdu 611731, China

**Keywords:** commuting, well-being, personality, gender, stress, Internet addiction

## Abstract

The focus of the present work was on the association between commuting (business and private), life satisfaction, stress, and (over-) use of the Internet. Considering that digital devices are omnipresent in buses and trains, no study has yet investigated if commuting contributes to the development of Internet addiction. Overall, *N* = 5039 participants (*N* = 3477 females, age *M* = 26.79, *SD* = 10.68) took part in an online survey providing information regarding their commuting behavior, Internet addiction, personality, life satisfaction, and stress perception. Our findings are as follows: Personality seems to be less suitable to differentiate between commuter and non-commuter groups, which is possibly due to commuters often not having a choice but simply must accept offered job opportunities at distant locations. Second, the highest levels of satisfaction were found with income and lodging in the group commuting for business purposes. This might be related to the fact that commuting results in higher salaries (hence also better and more expensive housing style) due to having a job in another city which might exceed job opportunities at one’s own living location. Third, within the business-commuters as well as in the private-commuter groups, females had significantly higher levels of stress than males. This association was not present in the non-commuter group. For females, commuting seems to be a higher burden and more stressful than for males, regardless of whether they commute for business or private reasons. Finally, we observed an association between higher stress perception (more negative attitude towards commuting) and Internet addiction. This finding suggests that some commuters try to compensate their perceived stress with increased Internet use.

## 1. Introduction

Commuting is a wide spread occurrence with millions of people afflicted around the globe [1]. Therefore, commuting behavior has the potential to influence both well-being and life satisfaction of a great part of the population either positive [2], where people regard travelling itself as enjoyable or negative [3], where they see it as a burden. The reasons to accept extended travel times are diverse. Some individuals commute to facilitate a better housing situation or to combine family and vocational goals [4]. Other reasons to commute might include promising career perspectives or financial incentives [5]. 

From an economical point of view, it seems logical that the costs and benefits of commuting should be at equilibrium to achieving decent levels of life satisfaction [3]. This means that increased costs of commuting (e.g., in terms of higher stress or lower well-being) should be compensated in some way, by higher benefits of the provided job opportunity, for example. Interestingly, this equilibrium is not always met and commuters are often willing to carry higher burdens than non-commuters. This has been coined as the ‘Commuting Paradox’ in the literature [5]. 

In the commuting literature physiological issues have also been investigated. A recent study examined the relation between commuting distance, cardiorespiratory fitness, and metabolic risk factors [6]. They found a negative association between commuting distance and physical activity (doing sports on a regular basis) as well as cardiorespiratory fitness and a positive association between commuting distance and Body-Mass-Index, waist circumference, and systolic/diastolic blood pressure. Another study from Norway [7] explored the association between long commutes and subjective health complaints. Again, those who reported longer travel times had more musculoskeletal pain and gastrointestinal problems. Moreover, commuters with a travel history of more than 10 years reported significantly more health complaints than those commuting for two years or less. The perceived level of stress should also be considered [8], since the commuting situation is often difficult to control (e.g., traffic jams, delays in public transport, bad weather conditions). This in turn has the potential to contribute to higher stress levels and frustration. Taking into account the link between health and well-being [9,10] the commuting situation should be associated with well-being. Interestingly, there is evidence that females suffer more under the burden of commuting than males. A study analyzing the effects of commuting on health with regard to gender [11] revealed that females with longer commuting times did seek medical advice and called in sick more often compared to males. An association between commuting and perceived higher stress levels could only be observed in females [12,13]. Presumably, the commuting situation, ceteris paribus, has a more adverse effect on females than males.

Aside from the relation between commuting and health/perceived stress, several studies investigated the direct link between commuting and life satisfaction. Here, the findings are mixed. An early study by Stutzer & Frey [3] concluded that commuting and life satisfaction are negatively related. Moreover, they demonstrated that commuting was characterized by rather low levels of positive effect, with a simultaneous fairly high negative effect. Similar findings were observed in another study investigating the commuting situation, salary, and life satisfaction [4]. In this work, commuting was negatively associated with overall life satisfaction but had no effect on the domains of life satisfaction, work and family. Furthermore, Lyons & Chatterjee [14] reported detrimental effects of commuting on stress, fatigue, and overall dissatisfaction. However, according to the authors, overall life satisfaction could even be increased if commuters can make their own decisions on how to use their travel time. If commuters experience these choices as worthwhile, a positive impact on life satisfaction could be the result [15]. In contrast to the studies mentioned so far, a positive relationship between commuting time and life satisfaction was found by Morris [16]. The authors observed such an association between commuting time and life satisfaction more strongly in rural areas and small cities. This association was visible only to a much weaker extent in large cities, most probably due to the higher degree of traffic congestions. Usually, traffic congestions are not controllable and therefore limit the individual’s self-determination. Subsequently, this could lead to a change in life satisfaction [17]. One study examined the association between life satisfaction and work commute [18]. The participants of this study reported mostly positive or neutral feelings during work commute and, consequently, a higher level of happiness. As possible reasons for this, the authors postulate that short work commutes could provide a buffer between work and private sphere, which in turn contributes to an increased level of well-being. For longer commutes, social and entertainment activities could counteract stress or boredom, as well as increase positive effects. 

It is worth noting that one possible reaction to stress might be the increased use of the Internet [19]. Since smartphones offer Internet access and are more often than not in people’s possession [20] it seems worthwhile to investigate the association between perceived stress (also in terms of attitude towards commuting) and Internet use [21] while commuting. If commuters show a negative attitude towards commuting and/or a high stress level one way to compensate for this could be an increased use of the Internet. As a growing number of researchers around the globe are currently investigating if problematic Internet use represents a societal problem (for an overview see Montag & Reuter [22]; Brand et al. [23]: I-PACE model; Petry & O’Brien [24]: Inclusion of Internet Gaming Disorder in Section III of DSM-5), the question arises if commuting itself also represents a vulnerability factor in becoming addicted to the Internet. Long commutes, in particular, could lead to excessive usage of digital channels. On the other hand, it is imaginable that commuting leads to less time spent on the Internet aside from commuting, because much of what needs to be done online has been already finished during the commute. In short, there is little awareness of studies investigating associations between Internet addiction and commuting. That is the reason why we also address this topic in this work. Internet addiction has been investigated for more than 20 years now [25,26] and much progress has been made to understand problematic Internet use. Although no consensus has been reached with respect to necessary symptoms of Internet addiction, symptoms such as preoccupation with the Internet, withdrawal symptoms when not being online, loss of control and problems in social/work life due to the overuse are of importance [27] Prevalence rates differ across the world, but in Germany (where the current investigated sample has been recruited) about 1% of the population is afflicted [28]. In the context of Internet addiction, it is necessary to distinguish between generalized (generalized pathological Internet use) and specific forms (e.g., excessive online gambling, shopping or social network use) of Internet addiction [29,30,31]. The present work focuses on unspecific tendencies towards Internet addiction in the realm of commuting.

Overall, an association between commuting and life satisfaction was found in many studies; however, the direction of this association is not trivial to understand. Moreover, it is noticeable that the reviewed research focuses on non-commuters vs. commuters, but they do not ask the question of whether people commute because of private (e.g., to see one’s own partner on the weekend) or business reasons (going to work and return back home). Reasons to commute could be an important factor because commuting may not always be considered as a burden [5], but as a fulfilling activity [2]. It is, therefore, conceivable that the motivation behind commuting or personality factors have an impact on the well-being of the commuters. Furthermore, there seems to be a gender specific effect in commuting, with females showing higher stress levels than males [13]. Therefore, in the present study (i) we investigated the association between commuting and life satisfaction in three separate groups: non-commuters, business commuters, and private commuters. In this context, we also examined the underlying personality structure in these groups since several studies reported associations between personality and life satisfaction [32,33,34,35] (see Supplementary Material for analyses on the associations of personality). Moreover (ii), we intended to replicate earlier findings about higher stress perception of females in commuting situations [11,12,13]. This time we also wanted to extend this in the realm of being a non-commuter, business-commuters, and private commuter. Finally (iii), we investigated the association between Internet addiction, life satisfaction, and stress (in relation to commuting). We expected a positive association between a negative attitude toward commuting (and high stress perception) and high Internet addiction.

## 2. Materials and Methods

For the present study, we asked participants to provide information via a specific designed online questionnaire covering various aspects of commuting. The online questionnaire could be filled in using any suitable device (e.g., tablet, smartphone, personal computer) with access to the Internet.

### 2.1. Participants

Overall *N* = 5039 participants (*N* = 3477 females) answered the online questionnaire and provided socio-demographic information, information on personality data and life satisfaction, as well as data concerning their commuting behavior. The mean age of the sample was 26.79 (*SD* = 10.68) ranging from 11 years to 98 years. Concerning educational training within the sample, the number of school leaving certificates was distributed as follows: a total of 31.8% had no school leaving certificate, 30.8% had a secondary school leaving certificate, 14.9% had a Baccalaureate-Diploma, and 22.5% had a university degree. Participation was voluntarily and completely anonymous. There was no monetary incentive, but upon completion of the questionnaire, all participants got a brief individual feedback on their personality profiles, life satisfaction, and Internet use (Internet addiction) based on the data provided. The local ethics committee of the Ulm University, Ulm, Germany approved the study, and all participants gave electronic consent prior to participation.

### 2.2. Materials

The data for the present study were gathered by means of an online questionnaire. In addition to collecting data on demographics, we requested information on personality (see Supplementary Material. For further information on the used personality questionnaire please refer to Rammstedt et al. [36] and John et al. [37]), life satisfaction, and Internet addiction. Furthermore, the participants gave information on their commuting status (none, business, private) and their (emotional) attitude towards commuting, particularly stress. To assess the overall attitude towards commuting, we asked the participants four questions (“Commuting does not matter to me” (item 1, inverse coding), “Commuting deteriorates my mood” (item 2), “Commuting deteriorates my quality of life” (item 3), and “Commuting stresses me” (item 4)). All items could be rated from 1 (“I do not agree at all”) to 5 (“I totally agree”). For the analyses, item 4 was analyzed both as a single item (to assess the participants stress level in relation to commuting) and also combined in the short four-item scale described above (to assess the overall ‘attitude towards commuting’; ATC). The scores of the four items are simply added up, after reversing the score of item 1. Cronbach’s alpha for the ATC scale was alpha = 0.85. 

Life satisfaction as one distinct part of subjective well-being, aside from positive and negative effect [38], was measured via questions retrieved from the German Socio-Economic Panel (SOEP) [39]. One section of the panel covers the current life situation within several areas, contributing to overall life satisfaction. For the purpose of this survey, we asked for the degree of satisfaction in the following areas: health, job, income, lodging, leisure, and overall satisfaction with life. Following a recommendation of the SOEP, the question for overall satisfaction with life was presented at the end of the life satisfaction questionnaire. This was done to avoid possible interference with specific domains of life satisfaction. It is important to note that overall life satisfaction is not a simple composite of the various domains of life satisfaction. In fact, all life satisfaction items are considered to be distinct, but also overlap to some extent (e.g., a person more satisfied with his leisure might have, as a consequence, a higher score on overall satisfaction). The items were answered, using a Likert scale, ranging from 0 (“completely dissatisfied”) to 10 (“completely satisfied”).

To gather data on Internet overuse we administered a short version of the Internet Addiction Test IAT [25], the short Internet Addiction Test (s-IAT) from [40]. This inventory consists of 12 items as opposed to the original version, which contains 20 items. The psychometric quality of the s-IAT has been considered to be of good effect [40]. The Cronbach’s alpha in our sample was high (alpha = 0.88). To more effectively assess the association between commuting and Internet use we asked an additional question; “because of commuting I use digital devices more often” (CMD). This could be rated from 1 (“I do not agree at all”) to 5 (“I totally agree”).

### 2.3. Procedure

Since the size of the total sample greatly relied upon the publicity of the online questionnaire, the study was introduced during interviews with several national radio and TV stations. This approach was taken to ensure high media coverage throughout Germany, and to avoid biases caused by the restriction of local samples. The audience was given a short introduction to the rationale of the study, combined with information on how to access the online questionnaire. Completed questionnaires were stored on servers and processed for further analyses.

### 2.4. Statistical Analysis

The statistical analyses were conducted using SPSS 22.0 for windows (IBM SPSS Statistics, Chicago, IL, USA). Differences in life satisfaction and personality variables for non-commuters, business commuters, and private commuters were investigated using ANOVAs, with the additional inclusion of gender effects. The associations between perceived stress, attitude towards commuting, Internet addiction, and life satisfaction were examined within the three groups of commuter type using Pearson correlations, as well as ANOVAs to test for gender effects.

## 3. Results

### 3.1. Data Cleaning and Descriptive Statistics

After inspection of the data, 25 participants were discovered to have provided conspicuous age information: A total of 20 participants reported an age of zero. Information on age was given using a slider ranging from 0 to 99 years, where 0 was the default value. Indicating an age of zero means that the participants did not provide any information on their age. Furthermore, we found 5 participants reporting an age between 1 and 9 years. Considering the rationale of the present study, it was uncertain as to whether or not these individuals indeed reported their real age and, if such was the case, were able to complete the questionnaire in a suitable way. Therefore, we decided to exclude these 25 participants, an overall (0.5% of the original sample; 18 commuting either for business or private plus 7 non-commuters) from the sample, which left an overall sample size of *N* = 5039 participants (*N* = 5064 before) for the analyses. Moreover, *N* = 409 participants reported both business-commuting and private-commuting, which forfeited their inclusion into one specific group. There were no further exclusions. We further investigated participants between 11 and 14, because at 15 years, persons are allowed to start working (with a vocational training). A closer inspection of participants with an age less than 15 showed that 28 persons (in our sample a total of 248 participants were younger than 15 years) reported to undertake business-commuting. This seems to be unusual considering the age of these participants. On the other hand, commuting to and from school (97.6% of the participants younger than 15 years reported to be still in school) is obligatory and therefore might be for some participants more connected to the term business rather than private-commuting. For that reason we did not exclude those participants from the sample. All variables were normally distributed and there were no outliers. Table 1 shows the descriptive statistics for the complete sample, and non-commuters (means, standard deviation, observed minimum and maximum values, skew and standard deviation of the skew) were calculated life satisfaction variables, Internet addiction (s-IAT), CSM (“Commuting stresses me”), and ATC (overall “attitude towards commuting”).

Please refer to Table 2 for the descriptive statistics of business-commuters and private commuters.

### 3.2. Association between Life Satisfaction and Commuting Status

**Figure 1:**
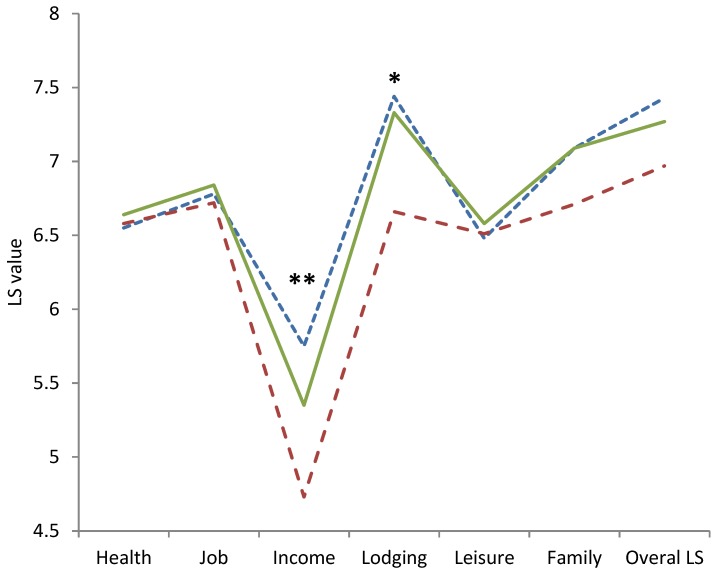
Life satisfaction values depending on commuting status: (BC) business-commuter (dotted blue line), (NC) non-commuters (solid green line), and (PC) private-commuters (dashed red line); * *p* = 0.01, ** (*p* < 0.001) significant differences between all three groups.

A one-way ANOVA showed a main effect of commuting status on income (*F*_(2,4627)_ = 9.53, *p* < 0.001; Levene’s test: *F*_(2,4627)_ = 7.40, *p* = 0.001), lodging (*F*_(2,4627)_ = 7.03, *p* = 0.001; Levene’s test: *F*_(2,4627)_ = 6.00, *p* = 0.003), and overall LS (*F*_(2,4627)_ = 3.41, *p* = 0.033; Levene’s test: *F*_(2,4627)_ = 4.61, *p* = 0.010). Since Levene’s test indicated the presence of heteroscedasticity, we used the Games-Howell test for post hoc analyses as recommended by Field & Miles [41]. The Games-Howell post-hoc analysis revealed significant differences between income scores of the non-commuting and business-commuting groups (*p* = 0.002; −0.39, 95% CI [−0.66, −0.12]), the non-commuting and private-commuting groups (*p* = 0.020; 0.63, 95% CI [0.08, 1.18]), and the business-commuting and private-commuting groups (*p* < 0.001; 1.02, 95% CI [0.43, 1.60]). For lodging scores, differences were found between non-commuters and private commuters (*p* = 0.004; 0.68, 95% CI [0.18, 1.18]) as well as between business-commuters and private-commuters (*p* = 0.002; 0.79, 95% CI [0.25, 1.32]). No effects on overall life satisfaction were found by post hoc analyses. To visualize our findings, please refer to Figure 1.

We also investigated these associations for gender effects. A one-way ANOVA showed a main effect of commuting status on income (*F*_(2,1420)_ = 4.32, *p* = 0.014; Levene’s test: *F*_(2,1420)_ = 9.06, *p* < 0.001) for males. Again, due to a positive Levene’s test, we used a Games-Howell test for the post hoc analysis and found a significant difference between non-commuting and business-commuting groups (*p* = 0.005; −0.57, 95% CI [−1.00, −0.15]) with higher income scores for the business-commuting group. For females, the analysis of variance revealed a main effect of commuting status on income (*F*_(2,3204)_ = 6.00, *p* = 0.003; Levene’s test: *F*_(2,3204)_ = 2.41, *p* = 0.090), ), lodging (*F*_(2,3204)_ = 4.81, *p* = 0.008; Levene’s test: *F*_(2,3204)_ = 3.47, *p* = 0.031), and overall LS (*F*_(2,3204)_ = 3.17, *p* = 0.042; Levene’s test: *F*_(2,3204)_ = 2.17, *p* = 0.114). A Games-Howell test revealed a significant difference for females between lodging scores of non-commuting and private-commuting groups (*p* = 0.015; 0.63, 95% CI [0.09, 1.17]) and of business-commuting and private-commuting groups (*p* = 0.006; 0.79, 95% CI [0.18, 1.40]). A generalized Tukey 2 post hoc test was used for income and overall life satisfaction since a Levene’s test did not indicate unequal variances. Females showed significant differences for income scores of the non-commuting and private-commuting groups (*p* = 0.007; 0.80, 95% CI [0.17, 1.43]) as well as for the business-commuting and private-commuting groups (*p* = 0.002; 1.03, 95% CI [0.32, 1.73]). Again, overall life satisfaction showed no significant result in the post hoc analysis. An independent-sample t-test provided significantly lower scores for health (*M_female_* = 6.56, *SD_female_* = 2.32; *M_male_* = 6.89, *SD_male_* = 2.25; *t*(3772) = 3.99, *p* < 0.001) and leisure (*M_female_* = 6.50, *SD_female_* = 2.37; *M_male_* = 6.78, *SD_male_* = 2.36; *t*(3772) = 3.31, *p* = 0.001) for females compared to males, but only in the non-commuting group.

The sample sizes used in the analyses for all groups considering commuting status and gender are summarized in Table 3. Age was not associated with commuting status (*r* = 0.003, *p* = 0.834).

### 3.3. Perception of Stress and Overall Attitude Towards Commuting

For the total sample, the CSM and ATC variable (“Commuting stresses me”; “Attitude towards commuting”) was negatively correlated with all life satisfaction variables in the business-commuters group but not in the private-commuters group. The same result was found when differentiating for gender; however, for both females and males all correlations between CSM/ATC and life satisfaction variables were negative (or not present) in the business-commuting group and nonexistent for females in the private-commuting group. In the private-commuting group we observed one significant negative correlation for males between CSM and overall LS (*r* = −0.39, *p* = 0.006, after Bonferroni correction for multiple testing; corrected alpha: 0.05/8=0.00625). All correlations are summarized in Table 4.

An ANOVA revealed that CSM scores were significantly higher for females (*M_female_* = 3.00, *SD_female_* = 1.17) compared to males (*M_male_* = 2.68, *SD_male_* = 1.20); *F*_(1674)_ = 12.13, *p* = 0.001) within the business-commuting group and the private-commuting group (*M_female_* = 3.05, *SD_female_* = 1.19; *M_male_* = 2.33, *SD_male_* = 1.19; *F*_(1178)_ = 12.59, *p* < 0.001). We observed no significant gender differences for the CSM score between business-commuting group, private-commuting group and non-commuting-group. However, ATC scores were significantly higher for females (*M_female_* = 11.09, *SD_female_* = 3.88) compared to males (*M_male_* = 10.19, *SD_male_* = 3.80); *F*_(1674)_ = 9.19, *p* = 0.003) within the business-commuting group and the private-commuting group (*M_female_* = 11.14, *SD_female_* = 3.81; *M_male_* = 9.13, *SD_male_* = 3.92; *F*_(1178)_ = 9.76, *p* = 0.002). No differences were observed between the business-, private-, and non-commuting groups (CSM: *F*_(2,1001)_ = 0.01, *p* = 0.994; ATC: *F*_(2,1001)_ = 1.58, *p* = 0.207).

### 3.4. Association of Internet Use, Attitude Towards Commuting, Stress, and Life Satisfaction

The s-IAT was positively associated with the attitude towards commuting (ATC) and stress perception (CSM) but only in the business-commuting group. For females, the association between CSM and IAT was *r* = 0.14, *p* = 0.005, and between ATC and IAT *r* = 0.12, *p* = 0.015. Males showed an association of *r* = 0.18, *p* = 0.003 between CSM and IAT score, and *r* = 0.18, *p* = 0.002 between ATC and IAT values. A positive correlation between ATC/CSM scores and CMD (“because of commuting I use digital devices more often”) was observed, again only in the business-commuter group. For females, the association between ATC and CMD was *r* = 0.24, *p* < 0.001 (males: *r* = 0.22, *p* < 0.001), while the association for CSM and CMD was *r* = 0.21, *p* < 0.001 (males: *r* = 0.22, *p* < 0.001). For females in the non-commuting group, the correlation between excessive Internet use and overall life satisfaction was *r* = −0.17, *p* < 0.001, while for males the correlation was *r* = −0.11, *p* < 0.001. For business and private commuters, we found significant associations between Internet addiction and life satisfaction only for females (business: *r* = −0.13, *p* = 0.009; private: *r* = −0.23, *p* = 0.009).

## 4. Discussion

The aim of this research was to extend already existing research on the association between personality, life satisfaction, and stress perception as a function of commuting status. No differences for personality were found between non-commuters, business-commuters, and private-commuters (see Supplementary Material as well as Baretta et al. [42] and Ruedl [43]). Significant associations were observed between income and lodging depending on commuting status. The business-commuting group showed the highest scores, while the private-commuting group the lowest scores for these areas of life satisfaction. As expected, stress perception was higher for females in the business-commuting group than males. In the private-commuting group the stress perception of females stayed high, whereas the stress perception of males decreased. However, a significant gender effect in relation to CSM/ATC was found only within the business and private-commuting group, i.e., not between the business- and private-commuting group or in the non-commuting group. A positive association was found between Internet addiction and both ATC and CSM, in the business-commuting group, regardless of gender. Moreover, a negative association between Internet addiction and overall life satisfaction (females and males) was found in the complete sample under investigation, as well as a negative association between Internet addiction and overall life satisfaction in the business and private-commuter groups, but for females only.

The association between life satisfaction and commuting [4,15,16,18] was also investigated. For two areas of life satisfaction, namely income and lodging, significant results were found. Interestingly, private-commuters showed lower and business-commuters higher life satisfaction scores than non-commuters for income and lodging. This finding is somewhat unexpected, as our rationale was that lower self-determination in the business-commuter group would lead to lower life satisfaction scores. However, one can infer that this emphasizes the distinct categorization between business-commuters and private-commuters. We discuss two possible explanations for this finding: First, self-determination might not be as a prominent predictor of life satisfaction as expected (at least in the context of commuting), and second, the presence or absence of a financial compensation in a commuting situation influences life satisfaction (life satisfaction scores for income shows the highest effects in our analyses). According to Erikson [44] and Newman et al. [17] self-determination represents an important factor that promotes higher life satisfaction scores. This, in fact, should lead to higher life satisfaction scores in the private-commuter group since within this group, the freedom of choice whether to commute or not should be higher as in the business-commuter group (as discussed above). The main reason why this is not the case and life satisfaction scores are higher for business-commuters could be due to the distinctiveness of the commuting situation: Business-commuters receive a financial compensation from the job, whereas private-commuters have only costs without any compensation. In this context, we propose that the monetary effect on life satisfaction, especially on income, could be much stronger than the influence of self-determination. Further analyses considering gender effects revealed that significant differences for lodging were present but only for females. Those who commuted for private reasons exhibited not only the lowest scores for income but also the lowest satisfaction scores in lodging. Considering that lodging is more important for females than males [45], it seems comprehensible that satisfaction scores for females in lodging are lowest for private commuters; the money they have spent on their private commute is no longer available to adequately furnish a home. Again, for business-commuting there is, at least, a financial compensation, which potentially enhances life satisfaction in this area.

Our findings regarding stress perception were mostly in accordance with previous findings asserting that females experience more stress in a commuting situation than males [8,46]. Interestingly, the level of perceived stress of females remained almost stationary regardless of whether they commuted for business or private reasons, whereas the stress level of males was much lower in the private-commuting group. Roberts et al. [13] proposed that the larger responsibility of females compared to males for daily household tasks could be the reason for higher stress levels of commuting females. In this case, it would be plausible that the reason for commuting (business or private) would have no influence on the female’s perception of stress because the outcome (possible neglect of household tasks due to commuting) is independent from the reason of commuting. The fact that significantly higher stress levels for females compared to males were present within the commuting groups, but not in the non-commuting group, further exacerbates findings concerning the detrimental effects of commuting, especially for females.

Also noticeable is the association between the attitude towards commuting/stress and Internet addiction [47]. Our findings point to a possible compensation of a negative attitude towards commuting by increased excessive Internet use. Interestingly, this association was only present in the business-commuter group and was independent of gender. Moreover, for the business-commuter group, again independent of gender, we observed a positive association between stress perception (attitude towards commuting) and CMD (“because of commuting I use digital devices more often”), which further supports this observation. The findings concerning the association between overall life satisfaction and Internet addiction, where females show more robust and stronger negative associations between Internet addiction and life satisfaction, also support earlier findings [48]. In this study, different thresholds have been suggested for females and males with respect to negative effects on life satisfaction because of high Internet use. Considering the present Internet addiction and commuting findings, one can denote forthcoming benefits from future studies examining what kinds of activities are done while commuting, and at what frequency. For instance, commuting time can be spent on work or learning new skills (e.g., via massive open online courses [49]) and should not be seen as fostering addictive tendencies towards digital technologies, per se. As previously mentioned, the diagnosis of Internet addiction depends on many criteria, but in the current research, it is merely the stressed commuter that is taken into account with regards to having higher Internet addiction tendencies.

The present study has strengths and also limitations worth noting. Firstly, using a cross-sectional design refrain us from causal inferences. It would be desirable to support the results of this study by replicating the main findings in longitudinal and experimental studies. Secondly, despite collecting a rather large sample, some group sizes (particularly the private-commuting group) used in the analyses are relatively small (see results section). Nevertheless, based on our considerations and findings we state that the discrimination between business and private commuters’ data can be used in a meaningful way to shed further light on the intriguing association between psychological (and even physiological) variables and commuting behavior. In addition to this, our data collection was accomplished by means of self-report questionnaires, which usually inherits a certain chance for biases. Future studies could minimize this potential shortcoming by using multiple approaches, such as peer reports or data logging to collect data. Furthermore, it should be noted that statements about the generalization of our findings should be done so with caution, since it is difficult to precisely pinpoint the locations of the respondents. On the other hand, the present study was promoted nationwide in Germany and relies on a fairly large sample, which adds to the validity of the findings. In addition, it is important to consider that the ratio of participants (non-commuters, business-commuters, private-commuters) simply reflects the response-rate of the participants from a nationwide promotional campaign. Naturally, this ratio may not be fully representative of the general population. Lastly, a concern of the present study was to learn more about the association between commuting, life satisfaction and Internet addiction. Therefore, we focused on the motivation to commute (private and business) rather than the association between duration/distance and life satisfaction, which has previously been investigated. Since there is evidence that some commuters possibly consider commuting as a fulfilling activity (independent of the duration/distance of the commute) this may be a consideration for further studies to shed light on.

## 5. Conclusions

In conclusion, the present study underlines the association between life satisfaction, stress perception, and commuting. Extending previous findings in literature, our results show that the reason to commute (business or private) has the potential to explain differences in life satisfaction variables, namely income and lodging. We also replicated previous findings concerning the gender specific role of stress in a commuting situation and its specific impact on females. Furthermore, we demonstrated that this association (in contrast to life satisfaction and commuting) did not vary, regardless of whether the commute was due to business or private reasons. Finally, the current study finds an association between the attitude towards commuting/stress and Internet addiction. Here, a more negative attitude towards commuting was associated with higher tendencies towards excessive use of the Internet. For future research, it would be beneficial to further investigate the association between the frequency and type of commuting behavior with Internet addiction. Moreover, strong insights can be provided by the use of applications to track activity on ubiquitous smartphones, while allowing access to specific questionnaires, and having GPS tracking activated. Essentially, this would mean combining self-reports with objective data directly collected from the smartphone. This research methodology will be complimentary to the emerging research discipline of Psychoinformatics [50]. Furthermore, this design would allow longitudinal studies to be conducted in an accessible and efficient way. Finally, future studies should aim to assess smartphone addiction, as inclinations of an overlap between Internet and smartphone addiction have been shown to exist [51,52], though they are not synonymous. Moreover, smartphones are clearly of high relevance in a commuter’s life, and recently, associations between smartphone addiction and lower productivity have also been reported [53].

## Figures and Tables

**Table 1:** Means and Standard Deviation, Minimum, Maximum and Skewness for life satisfaction variables, and Internet variables for the complete sample (CS) and non-commuters (NC).

Variables	Min	Max	Mean	SD	Skewness	SD
Overall satisfaction	0	10	7.28/7.27	2.19/2.20	−1.06/−1.05	0.03/0.04
Health	0	10	6.62/6.65	2.30/2.30	−0.71/−0.69	0.03/0.04
Job (*N* = 4616/3401)	0	10	6.81/6.82	2.42/2.43	−0.86/−0.87	0.03/0.04
Income	0	10	5.38/5.36	2.96/3.00	−0.28/−0.26	0.03/0.04
Lodging	0	10	7.30/7.33	2.53/2.54	−1.04/−1.05	0.03/0.04
Leisure	0	10	6.55/6.58	2.38/2.37	−0.56/−0.56	0.03/0.04
Family	0	10	7.07/7.10	2.60/2.60	−0.90/−0.90	0.03/0.04
CSM (*N* = 1413)	1	5	2.86/2.86	1.20/1.20	−0.05	0.07
CMD	1	5	2.85/3.11	1.45/1.32	−0.02/−0.27	0.07/0.20
ATC	4	20	10.72/11.29	3.85/3.77	0.16/0.11	0.07/0.20
s-IAT	12	60	27.01/27.06	8.37/8.45	0.74/0.72	0.03/0.04

(*N* = 5039/3774, CS/NC), Internet use (s-IAT), CSM (“Commuting stresses me”), ATC (overall “attitude towards commuting”), and CMD (“because of commuting I use digital devices more often”).

**Table 2:** Means and Standard Deviation, Minimum, Maximum, and Skewness for life satisfaction variables, and Internet variables for business-commuters (BC) and private commuters (PC).

Variables	Min	Max	Mean	*SD*	Skewness	*SD*
Overall satisfaction	0	10	7.43/6.97	2.08/2.52	−1.17/−1.00	0.09/0.18
Health	0	10	6.55/6.58	2.22/2.36	−0.75/−1.00	0.09/0.18
Job (*N* = 663/153)	0	10	6.78/6.72	2.38/2.31	−0.80/−0.79	0.10/0.20
Income	0	10	5.75/4.73	2.71/3.05	−0.43/−0.16	0.09/0.18
Lodging	0	10	7.44/6.66	2.39/2.77	−1.19/−0.76	0.09/0.18
Leisure	0	10	6.48/6.51	2.32/2.52	−0.60/−0.72	0.09/0.18
Family	0	10	7.09/6.71	2.55/2.76	−0.91/−0.78	0.09/0.18
CSM	1	5	2.86/2.86	1.19/1.23	1.19/−0.14	0.09/0.18
CMD	1	5	2.66/2.94	1.48/1.40	0.19/−0.11	0.09/0.18
ATC	4	20	10.71/10.61	3.87/3.93	0.19/0.12	0.09/0.18
s-IAT	12	60	26.30/27.77	7.71/8.49	0.75/0.68	0.09/0.18

(*N* = 676/180, BC/PC). Internet use (IAT), CSM (“Commuting stresses me”), ATC (overall “attitude towards commuting”), and CMD (“because of commuting I use digital devices more often”).

**Table 3:** Sample sizes depending on commuting status and gender.

Sample	B/P *-Commuting	Non-Commuting	Business-Commuting	Private-Commuting
Complete *N* = 5039	409	3774	676	180
Female *N* = 3477	270	2684	391	132
Male *N* = 1562	139	1090	285	48

* B/P: Business and Private.

**Table 4:** Correlations between CSM (“Commuting stresses me”), ATC (“Attitude towards commuting”; 4 item scale), and life satisfaction variables.

Commuting Status	Health	Job	Income	Lodging	Leisure	Family	Overall LS
BC	−0.11 **/−0.13 **	−0.25 **/−0.28 **	−0.22 **/−0.22 **	−0.16 **/−0.18 **	−0.21 **/−0.26 **	−0.10 **/‒0.16 **	−0.14 **/−0.19 **
PC	−0.02/−0.01	−0.06/−0.10	0.01/0.03	−0.10/−0.11	−0.07/−0.09	0.01/0.02	−0.11/−0.11
BC (male)	−0.15 */−0.18 **	−0.28 **/−0.33 **	−0.22 **/−0.23 **	−0.12 */−0.15 *	−0.24 **/−0.34 **	−0.14 */−0.23 **	−0.17 **/−0.25 **
BC (female)	−0.07/−0.09	−0.24 **/−0.26 **	−0.21 **/−0.20 **	−0.19 **/−0.20 **	−0.19 **/−0.20 **	−0.07/−0.12 *	−0.14 **/−0.16 **
PC (male)	−0.14/−0.14	−0.41 */−0.39 *	0.03/0.10	−0.17/−0.03	−0.14/−0.01	−0.03/0.16	−0.39 **/−0.31 *
PC (female)	0.02/0.03	0.03/−0.04	0.04/0.05	−0.08/−0.15	−0.03/−0.11	0.00/−0.05	−0.03/−0.05

BC = business-commuting, PC = private commuting, CSM/ATC; * *p* < 0.05, ** *p* < 0.001.

## Supplementary Materials

The following are available online at www.mdpi.com/1660-4601/14/10/1176/s1, Table S1: Means and Standard Deviation, Minimum, Maximum, and Skew of personality variables for for the complete sample (CS) and non-commuters (NC), Table S2: Means and Standard Deviation, Minimum, Maximum, and Skew of personality variables for business-commuters (BC) and private commuters (PC).
