# Supplementary material for: Commuting, Life-Satisfaction and Internet Addiction
Source: Int J Environ Res Public Health. 2017 Oct 5;14(10):1176. doi: 10.3390/ijerph14101176 (PMC5664677; doi:10.3390/ijerph14101176)
Supplement: Supplementary file 1 [file ijerph-14-01176-s001.pdf]

Association between Personality and Commuting Status

Personality data was collected using the Big Five Inventory 10 (BFI-10) [38], which individually measures the five factors of personality—neuroticism, extraversion, agreeableness, openness, and conscientiousness. This questionnaire consists of ten items (2 for each personality dimension) and is relying on a Likert scale ranging from, 1 ("disagree strongly") to 5 ("agree strongly"). Originally, the BFI-10 was developed on the basis of the Big Five Inventory-44 [39] and intensively tested by Rammstedt et al. [36]. Substantial correlations between these two questionnaires were presented in this work, ranging from $r = 0.74$ (agreeableness) to $r = 0.89$ (extraversion) with an acceptable test-retest stability for the BFI-10 of $r = 0.75$ (BFI-44: $r = 0.83$). One can, therefore, denote that the BFI-10 can be used efficiently in various research settings.

We analyzed the association between personality and commuting status between all commuting groups also considering potential gender effects. No significant effects were found. Between the groups a one way ANOVA rendered the following results: Neuroticism $F_{(2,4627)} = 2.74$, $p = 0.065$; Extraversion $F_{(2,4627)} = 1.85$, $p = 0.158$; Openness $F_{(2,4627)} = 0.73$, $p = 0.480$; Agreeableness $F_{(2,4627)} = 0.11$, $p = 0.892$; Conscientiousness $F_{(2,4627)} = 2.57$, $p = 0.076$. For males the results were as follows: Neuroticism $F_{(2,1420)} = 0.31$, $p = 0.732$; Extraversion $F_{(2, 1420)} = 1.67$, $p = 0.189$; Openness $F_{(2, 1420)} = 0.26$, $p = 0.774$; Agreeableness $F_{(2, 1420)} = 0.22$, $p = 0.806$; Conscientiousness $F_{(2, 1420)} = 0.59$, $p = 0.552$ and for females Neuroticism $F_{(2,3204)} = 0.72$, $p = 0.486$; Extraversion $F_{(2, 3204)} = 0.12$, $p = 0.885$; Openness $F_{(2, 3204)} = 2.13$, $p = 0.119$; Agreeableness $F_{(2, 3204)} = 1.46$, $p = 0.232$; Conscientiousness $F_{(2, 3204)} = 0.76$, $p = 0.469$ (post-hoc tests showed also no significant result). For descriptive information on personality please refer to Tables S1 and S2.

Table S1. Means and Standard Deviation, Minimum, Maximum, and Skew of personality variables for the complete sample (CS) and non-commuters (NC).

Variables	Min	Max	Mean	SD	Skew	SD
Neuroticism	1	5	2.93/2.94	0.91/0.91	0.09/0.09	0.03/0.04
Extraversion	1	5	3.48//3.49	0.96/0.96	-0.31/-0.31	0.03/0.04
Openness	1	5	3.38/3.37	0.98/0.99	-0.17/-0.16	0.03/0.04
Agreeableness	1	5	3.10/3.11	0.79/0.79	-0.12/-0.11	0.03/0.04
Conscientiousness	1	5	3.34/3.34	0.82/0.83	-0.02/-0.03	0.03/0.04

(N = 5039/3774, CS/NC).

Table S2. Means and Standard Deviation, Minimum, Maximum, and Skew of personality variables for business-commuters (BC) and private commuters (PC).

Variables	Min	Max	Mean	SD	Skew	SD
Neuroticism	1	5	2.85/2.99	0.88/0.93	0.05/0.23	0.09/0.18
Extraversion	1	5	3.42/3.51	0.98/0.91	-0.35/-0.20	0.09/0.18
Openness	1	5	3.42/3.38	0.99/0.96	-0.24/0.01	0.09/0.18
Agreeableness	1	5	3.04/3.15	0.78/0.84	-0.07/-0.22	0.09/0.18
Conscientiousness	1	5	3.33/3.31	0.76/0.82	-0.01/-0.17	0.09/0.18

(N = 676/180, BC/PC).

In our analyses we first investigated possible differences in personality structure depending on commuting status. No differences were observed, which suggests that people who commute are not driven to do so because of a unique personality structure like, for example, individuals with high novelty seeking scores who prefer playing a risky sport [42,43]. Often, there is little choice and one simply must commute in order to earn money, get a job, or for the upkeep of social activity, even if commuting means the deterioration of more intrinsic and personal processes [5]. Thus, practical constraints seem to be the crucial factor in determining commuting behavior and not personality. An important requirement to detect the impact of a personality structure is the presence of alternative options and the individual's freedom of choice to make a decision according to their specific personality. If this simply is not the case and practical constraints are prevalent, a specific personality structure as a factor of commuting status will not be obtainable.
